# A realistic phantom of the human head for PET-MRI

**DOI:** 10.1186/s40658-020-00320-z

**Published:** 2020-08-05

**Authors:** Johanna Harries, Thies H. Jochimsen, Thomas Scholz, Tina Schlender, Henryk Barthel, Osama Sabri, Bernhard Sattler

**Affiliations:** 1 Department of Nuclear Medicine, Leipzig University Hospital, Liebigstr. 18, Leipzig, Germany; 2 Department of Radiation Safety and Medical Physics, Medizinische Hochschule Hannover, Carl-Neuberg Straße 1, Hannover, Germany

**Keywords:** PET-MRI, Attenuation correction, Brain phantom

## Abstract

**Background:**

The combination of positron emission tomography (PET) and magnetic resonance imaging (MRI) (PET-MRI) is a unique hybrid imaging modality mainly used in oncology and neurology. The MRI-based attenuation correction (MRAC) is crucial for correct quantification of PET data. A suitable phantom to validate quantitative results in PET-MRI is currently missing. In particular, the correction of attenuation due to bone is usually not verified by commonly available phantoms. The aim of this work was, thus, the development of such a phantom and to explore whether such a phantom might be used to validate MRACs.

**Method:**

Various materials were investigated for their attenuation and MR properties. For the substitution of bone, water-saturated gypsum plaster was used. The attenuation of 511 keV annihilation photons was regulated by addition of iodine. Adipose tissue was imitated by silicone and brain tissue by agarose gel, respectively. The practicability with respect to the comparison of MRACs was checked as follows: A small flask inserted into the phantom and a large spherical phantom (serving as a reference with negligible error in MRAC) were filled with the very same activity concentration. The activity concentration was measured and compared using clinical protocols on PET-MRI and different built-in and offline MRACs. The same measurements were carried out using PET-CT for comparison.

**Results:**

The phantom imitates the human head in sufficient detail. All tissue types including bone were detected as such so that the phantom-based comparison of the quantification accuracy of PET-MRI was possible. Quantitatively, the activity concentration in the brain, which was determined using different MRACs, showed a deviation of about 5% on average and a maximum deviation of 11% compared to the spherical phantom. For PET-CT, the deviation was 5%.

**Conclusions:**

The comparatively small error in quantification indicates that it is possible to construct a brain PET-MRI phantom that leads to MR-based attenuation-corrected images with reasonable accuracy.

## Background

The combination of positron emission tomography (PET) and magnetic resonance imaging (MRI) (PET-MRI) is a very promising hybrid imaging technique which combines the high soft tissue contrast of MRI with the high functional sensitivity of PET [1]. However, the major technological challenge in PET-MRI is the MRI-based attenuation correction (MRAC) [2]. To quantify metabolic or physiological function by means of radiotracer distribution, kinetics, and radioactivity concentrations in PET, attenuation by the tissue of the patient has to be accounted for [3]. The attenuation of the annihilation photons, which are measured by PET, depends on the electron density of the traversed tissue. MRI, on the other hand, is based on measuring the magnetization of the atomic nuclei. These two tissue properties are not directly related to each other [1]. For MRAC, several methods are available that provide routine AC (cf. Table 1).

**Table 1:** MR-based attenuation correction methods with their sequence parameters and post-processing strategies

MRAC	Sequence	TE [ms]	TR [ms]	Post-processing	Reference
DIXON	Dixon	1.23/2.46	3.96	Fat/water segmentation (built-in)	[11]
BRAIN HiRes	Dixon	1.28/2.51	4.14	Fat/water/bone segmentation (built-in)	*Segbone* in [28]
UTE	UTE	0.07/2.46	4.64	Bone/tissue segmentation (built-in)	[29]
RESOLUTE	UTE	0.07/2.46	4.64	Continuous *μ*-map (offline)	[15]
PSEUDO-CT	MPRAGE	2.53	1900	Atlas-based (offline)	[16]
pCT	MPRAGE	2.53	1900	Atlas-based (offline)	[17], optimized
					for accuracy

Usually, phantoms are used to validate the quantitative results of imaging systems, for example in PET [4] or in single photon emission computed tomography (SPECT) [5]. However, it is usually not possible to use these general-purpose phantoms in PET-MRI to evaluate the accuracy of measuring tracer activity concentration [6, 7]. In order to obtain useful quantitative results, additional CT-based attenuation correction is required for current phantoms [8]. Hence, an evaluation of clinical MRAC procedures is currently only possible on the basis of patient examinations because building a phantom that resembles the human body with respect to both its MRI and PET properties is challenging for several reasons: Firstly, material with protons accessible by MRI has to be used. This requirement rules out most solid objects with a broad proton peak in the nuclear magnetic resonance spectrum. Secondly, the human body contains different tissue types with a wide range of proton relaxation rates (from several ms in bone to the order of seconds in fluids) which have to be imitated by a phantom. Thirdly, the imitations have to match the attenuation of PET photons [9]. Moreover, as most MRAC methods assume a particular anatomical configuration of tissue types (e.g., in atlas-based methods), the phantom has to resemble the geometry of the human head for these methods.

The current study presents a head-shaped phantom made of different materials imitating MRI- and PET-specific properties of tissue in the human head. Using this phantom, the results from various MRAC methods were compared to assess whether the phantom is a suitable surrogate of the human head for PET-MRI.

## Method

### Tissue substitutes and their properties

Materials used in this study were investigated with respect to their attenuation of gamma ray photons and MR properties. The attenuation properties were determined on the basis of a transmission measurements using a stand-alone PET system (ECAT-EXACT-HR +, Siemens Healthcare, Germany) equipped with Ge-68 line sources. The PET-MRI system (Biograph mMR with E11P software, Siemens Healthcare, Germany) assigns a predefined attenuation coefficient to the different tissue types in most AC methods. Therefore, for the purpose of this study, the substitutes should have approximately the same linear attenuation coefficients, *μ*, as assumed by the PET-MRI system, which are listed in Table 2.

**Table 2:** Attenuation properties of the tissue substitutes as expected by the MRACs of the system (*μ*_desired_) and measured using a PET transmission scan (*μ*_measured_) and CT(*μ*_CT_)

Tissue	*μ*_desired_[c*m*^−1^]	Substitute	*μ*_measured_[c*m*^−1^]	*μ*_CT_[c*m*^−1^]
Bone	0.151 cm ^−1^	Gypsum plaster	0.1501 ± 0.0013	0.1492 ± 0.0006
Brain	0.1 cm ^−1^	Agarose gel	0.0938 ± 0.0004	0.0963 ± 0.0003
Fat	0.0854 cm ^−1^	Silicone	0.0846 ± 0.0015	0.1030 ± 0.0010

The MR properties were determined on the basis of Dixon- and ultra-short echo time (UTE)-based MRAC sequences. The Dixon sequence uses the separate water and lipid resonance signals by phase-sensitive MRI and provides separate water and fat images [10]. For MRAC, these maps are then used in a segmentation to create a *μ*-map consisting of regions with water-dominated and fat-dominated tissue [11]. On the basis of the Dixon data, template-based methods are possible where a model of the skull is non-rigidly registered to the subject (BRAIN HiRes in Table 1). On the basis of the separate images, it was also possible to determine whether the imitations of brain and fat tissue were correctly identified.

UTE pulse sequences are designed to deal with the difficulty of acquiring MR signal from protons with short $T^{\star }_{2}$, such as those of bone [12, 13]. Zero echo time (ZTE) sequences [14] are a derivative of this approach. For MRAC, UTE/ZTE is combined with data acquired with TE on the order of several ms so that the difference yields the MR signal of bone which is then used in a segmentation to create a *μ*-map consisting of bone and soft tissue. The UTE data can also be used in region-specific optimization of continuous linear attenuation coefficients based on UTE (RESOLUTE) to provide more sophisticated *μ*-maps [15].

Another possibility to create a *μ*-map based on MR data is to use high-resolution anatomical data in an image registration based on morphological similarities which yields a coregistered CT-based *μ*-map derived from training data of a cohort of patients [16, 17].

#### Bone

To imitate bone with respect to its MRI properties, a material was required which has a strong water signal and a short $T^{\star }_{2}$ on the order of ms to imitate the pore water fraction of cortical bone. Preliminary tests prior to this study have shown that gypsum plaster, which was bond with water and then kept wet by storing it permanently in water, is very well suited for this purpose. Recently, this approach has also been suggested elsewhere [18]. The mixing ratio (gypsum to liquid) of 10:7 turned out to be useful for further processing steps due to its fluent consistency. It was easy to pour it into the negative mold (see below), and it hardened within a few hours. The attenuation of 511 keV annihilation photons was regulated by the addition of iodine. With an iodine concentration of 92 mg/ml corresponding to 23% iodine-containing CT contrast agent (Imeron 400 MCT, Bracco Imaging, Konstanz, Germany), it was possible to adjust the desired attenuation coefficient (cf. Table 2).

Using PET-MRI, gypsum was examined using the UTE sequence [13]. In order to determine whether the materials deliver MR signals similar to those of bone, the intensities in the image data of the two echo times were used to calculate effective relaxation rate $R^{\star }_{2}=1/T^{\star }_{2}$. The reference values were determined from patient data. For this purpose, the UTE images of twenty clinical routine patients were analyzed. The cortical bone of each patient was segmented according to the UTE-based *μ*-map created by the PET-MRI system. Within this mask, mean values and standard deviations (SD) were calculated with the program ITK Snap [19]. Using the determined intensities of the two echo times of the UTE sequence, $R^{\star }_{2}$ of the bone of the individual patients was calculated.

#### Fat

The MR signal of adipose tissue is mainly caused by protons in methylene groups (C*H*_2_) and differs from that of water protons in its chemical shift. This difference is typically used in a Dixon sequence to discriminate between adipose and non-adipose tissue. Simple one-component sanitary silicone was used to imitate adipose tissue. Silicone is a polysiloxane having methyl groups (C*H*_3_) which exhibit a chemical shift similar to that of fat [20, 21].

In order to determine the suitability of this material, it was examined using the built-in Dixon sequence of the PET-MRI which is also used for MRAC. Ideally, the material should only be visible in the fat image of the Dixon sequence and, hence, identified as fat in the *μ*-map. In addition to the Dixon sequence, the material was examined with the UTE sequence, where the image intensity of the material should be almost identical for both echo times. Hence, it should be identified as soft tissue instead of bone or air.

#### Brain

Agarose gel was used to imitate brain tissue [22–24]. To obtain relaxation times similar to those of human brain tissue, 1 mM NiC*l*_2_ in water was mixed with 1.6% agarose powder.

### Creating the phantom

**Fig. 1:**
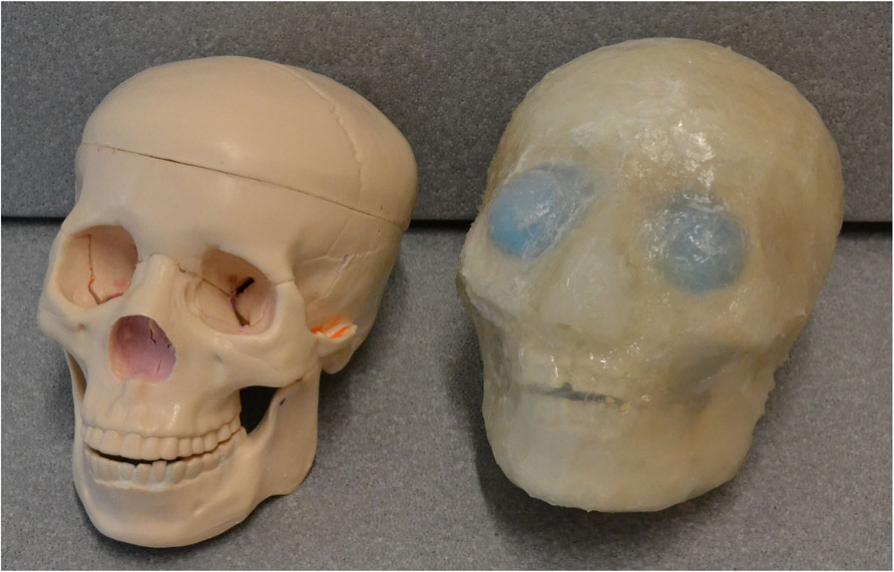
Commercial skull model (left) which served as a template for creating the bones, and final phantom (right)

In order to bring the gypsum plaster into anatomical shape, an existing plastic skull model was used (Fig. 1). The model consists of the skull base, the skull calotte, and the mandible. These three parts can be separated from each other. Inner and outer negative molds were required to bring all three parts of the plaster into anatomical shape. To create the negative molds, the three parts of the model were separated, and then, an inner and outer negative form was created for each of the three parts. Silicone rubber consisting of two components, which cure after mixing, was used to create the mold. Using rubber as an impression material has the advantage that it can be easily and non-destructively removed from the model so that the negative mold can be reused.

When filling the negative mold with plaster, care was taken that the inner negative mold was positioned in the center of the outer negative mold. The shapes were marked for correct placement. The model was inserted into the negative molds for this purpose. In this position, the inner and outer molds were correctly aligned to each other. With the help of markings on the front, back, and sides of the skull, the molds were aligned with each other when filling with plaster.

Like the human head, the skull base of the phantom has an opening (foramen magnum) in the area of the posterior fossa. This opening was used to pour in the substitute for brain mass and to fill the phantom with radioactive solution. The possibility to place radioactive solution in the phantom was realized by a 50-ml flask (50-mm diameter, 1-mm wall thickness) made of polymethylpentene. This flask is wider than the hole. For this reason, it was placed inside the skull before the bone imitations were joined together. The neck of the flask was pushed through the foramen magnum from the inside and marked on the neck. The marking is necessary to place the flask approximately in the center of the brain while filling the skull with agarose (see below). The bottleneck protrudes from the bottom of the phantom so that it can be filled from outside. The flask can be closed with a stopper.

Between the two halves of the skull was a small gap due to unevenness, which was closed with a plaster mixture when joining both halves. Since the new plaster did not bind well with the old one, the bonding area was stabilized with fabric tape and epoxy resin. For this purpose, the fabric tape was placed around the skull at the point of fracture and coated with epoxy resin. The epoxy resin forms a thin film on the plaster and is not visible on the MRI or PET data due to its low expansion, and hence, it is assumed that it does not significantly attenuate the annihilation photons.

For the imitation of brain tissue, NiC*l*_2_ and agarose were dissolved in water and heated during constant stirring. The liquid mixture was then filled into the imitation of the bone and hardened as it cooled down. It contracted slightly during this process. Hence, the inside of the skull was not filled completely at first. Therefore, in a second step, the remaining cavities were filled again. Openly accessible areas were filled directly as before. Air inclusions inside the phantom were made freely accessible with a small drill and filled using a syringe.

The scalp as well as the human face consists mainly of adipose soft tissue. Therefore, these areas of the phantom were modeled using silicone. Before the silicone was applied, water-filled balloons were placed in the eye sockets as ocular balls. A thin layer of one-component silicone was applied to the top of the skull imitating scalp skin. The mandible was attached to the phantom with the help of a continuous silicone layer.

The nose and other facial features were also modeled using silicone. The oral cavity was cut out and later filled with agarose gel because, genuinely, it consists mainly of mucous membranes with high water content. Hoses were used to introduce air cavities as trachea and esophagus. Once the gel has hardened in the oral cavity, the phantom was closed with one-component silicone in the lower area as well. When applying the silicone, care was taken to avoid gaps or holes and to avoid the plaster coming into contact with air so that it does not dry out.

### Acquisition

The practicability of the phantom with respect to the comparison of MRACs was checked as follows: The phantom-inserted flask and a spherical phantom (hollow sphere made of acrylic glass, 160-mm diameter, 1-mm wall thickness) were filled from the same stock solution (50 kBq/ml F18-FDG, mixed in advance). The spherical phantom served as a reference with negligible error in MRAC and without partial volume effect in the central region. The attenuation coefficient of acrylic glass is *μ*_acryl_=0.101 c*m*^−1^ [25] which is almost identical to that of water. The DIXON method was selected among the different MRAC methods because it produced a *μ*-map with a diameter identical to the outer diameter of the sphere which was verified manually by counting the voxels along a line through the center of the sphere. The activity concentration was measured in both vessels by PET-MRI using the MRACs listed in Table 1 and by PET-computed tomography (PET-CT).

For PET-MRI, the PET data was acquired for 4 min and reconstructed into a 256 × 256 matrix (voxel size, 2.32 × 2.32 × 2.03 mm ^3^) using the built-in 3D ordered subset expectation maximization (OSEM) algorithm with 3 iterations, 21 subsets and a 3-mm Gaussian filter.

On the PET-CT system (Biograph 16, Siemens Healthcare, Germany), the PET data was acquired over 4 min and reconstructed into a 168 × 168 matrix (voxel size, 4.07 × 4.07 × 3.0 mm ^3^) using 3D OSEM with 4 iterations, 8 subsets, and a 5-mm Gaussian filter. CT data was acquired with 37-mAs current-exposure time product and 120-kV peak tube potential. It was converted to the corresponding *μ*-maps by a bilinear transformation [26]. Representative regions of interest (ROIs) were placed in bone (behind the nose), brain tissue, and fat/silicone (bottom of the phantom), respectively.

All measurements were performed six times to estimate the stochastic error.

### Analysis

The Object-oriented Development Interface for NMR [27] was used for all processing steps (URL: http://od1n.sourceforge.net). The PET data was decay-corrected to a common reference time before comparison. In order to reduce partial volume effects, central ROIs in the flask and the spherical phantom (volumes of 22 ml and 1338 ml, respectively) with a minimum gap of approximately 6 mm between ROI and vessel rim resulting in negligible partial volume effects were drawn manually. By averaging over these ROIs and by comparing activity concentrations of the flask and the spherical phantom in these regions during the same session using an identical setup, the effectiveness of the MRACs was studied free of other influences, for instance, as introduced by an external dose calibrator.

## Results

The reference relaxation value for cortical bone as determined from the patient data was $R^{\star }_{2,\text {patients}}=(0.28~\pm ~0.04)~{\mathrm {ms^{-1}}}$ (mean and standard deviation over patients). The MRI data of the gypsum with the mixing ratio mentioned above provided an identical value of $R^{\star }_{2,\text {gypsum}}=(0.28~\pm ~0.08)~{\mathrm {ms^{-1}}}$. Table 2 lists the measured attenuation coefficients of the tissue substitutes. A photograph of the final phantom is shown in Fig. 1.

The one-component silicone had a reduced signal during the second echo time in contrast to the first echo time in the UTE sequence corresponding to $R^{\star }_{2,\text {silicone}}$ of roughly 0.25 m*s*^−1^. Hence, relaxation is stronger (shorter $T^{\star }_{2}$) than in adipose tissue. However, when creating the *μ*-map, the silicone was correctly segmented as soft tissue.

**Fig. 2:**
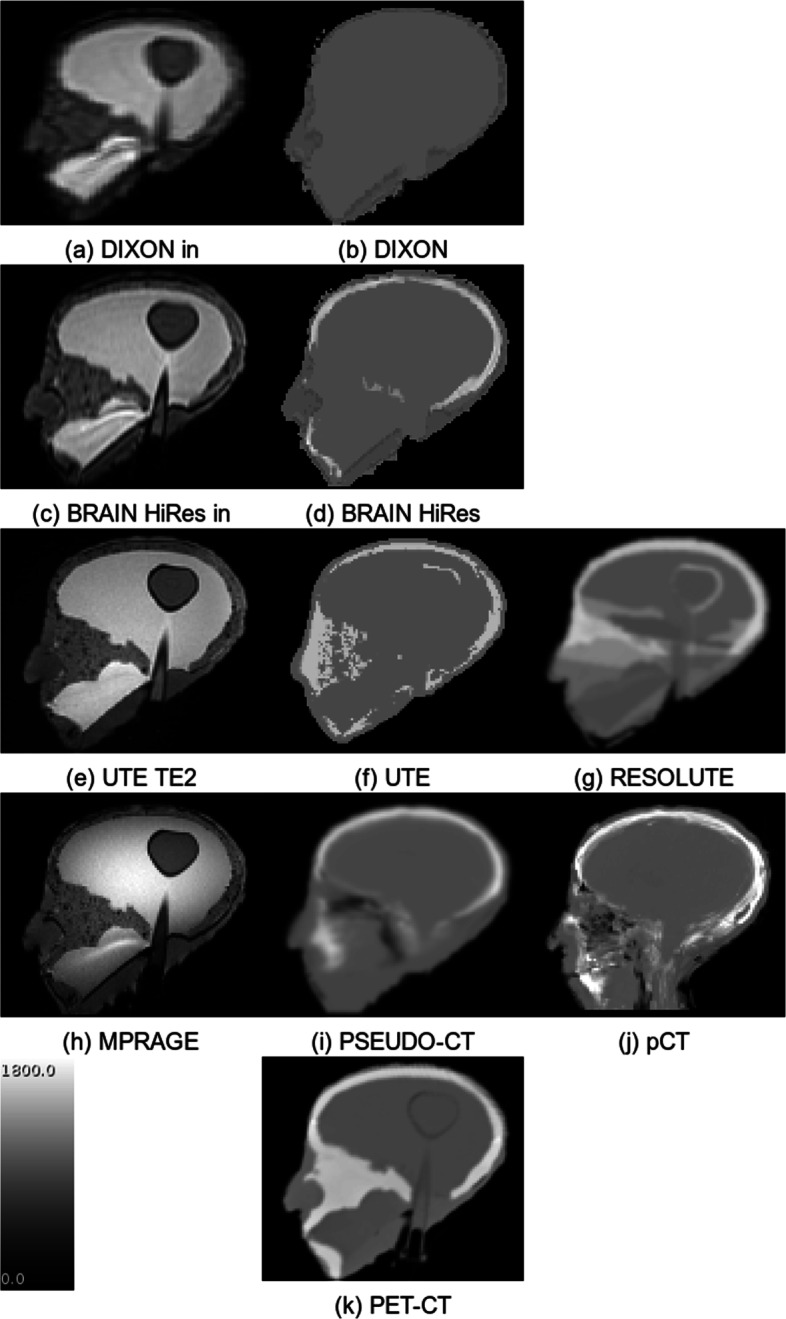
MRI data (leftmost column) and *μ*-maps derived from it (other columns). **a**, **c** The magnitude of the in-phase Dixon data and **e** the second echo of the UTE sequence. The *μ*-maps are labeled the same as in Table 1. For comparison, **k** displays the *μ*-map derived from the CT data of the PET-CT examination

**Fig. 3:**
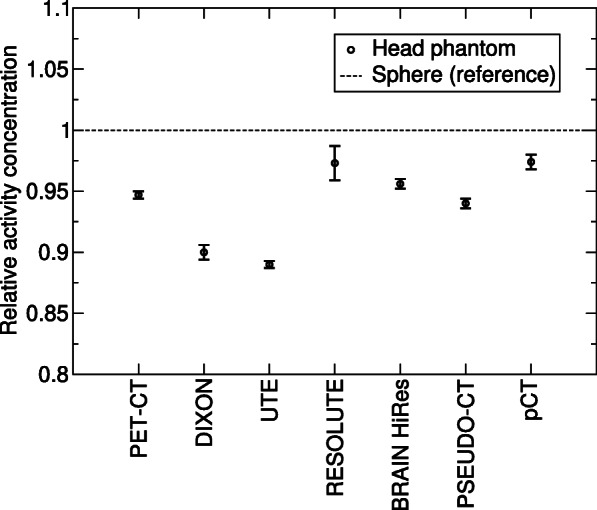
Comparison of the measured activity concentration between the spherical phantom and the created PET-MRI phantom. The error bar is the standard deviation calculated over six repeated measurements

The *μ*-maps created by the MRACs and PET-CT are summarized in Fig. 2. The maps show that, in general, the phantom was correctly represented and that, depending on the capabilities of the MRAC method, the segmentation of tissue types was successful for all MRAC methods. The quantitative results of the comparison between head phantom and water sphere are shown in Fig. 3. The activity concentration in the brain, which was determined by PET-MRI and various MRAC methods, showed an average deviation of about 5% and a maximum deviation of 11% compared to the spherical phantom. For PET-CT, the deviation was 5%.

## Discussion

The phantom imitated the human head in sufficient detail with respect to the attenuation and MR properties. All tissue types including bone were detected so that the phantom-based comparison of the quantification accuracy of PET-MRI became possible. The comparison to data from actual patients [28] shows a similar global behavior of the AC methods. In particular, when comparing Fig. 3 of this study with Fig. 3 in [28] and by taking into account that the latter uses PET-AC as the reference (instead of a tracer-filled sphere as in this study) and by assuming that genuine PET-CT and CT-based AC of PET-MRI yield the same results, the following similarities can be observed: DIXON and UTE underestimate the activity concentration whereas BRAIN HiRes (corresponding to *Segbone* in [28]), PSEUDO-CT (*Boston*), and pCT (*UCL*) show good correspondence. For RESOLUTE, it is known that using the algorithm with the current software version of the PET-MRI system (E11P) leads to results inferior to those obtained in combination with the previous software (B20P). This is due to modifications in the vendor-provided UTE sequence that is the basis for RESOLUTE.

There are several potential sources of error with the current phantom: The attenuation of the walls of the flask inside the phantom and the thin layer of glass-fiber-reinforced epoxy resin are not taken into account in the MRAC. Moreover, the wall of the flask is partly recognized as bone in UTE and RESOLUTE (cf. Fig. 2) which can lead to overestimation of tracer concentration within the flask. However, it can be assumed that this effect is small compared to the following sources of error caused by the MRAC methods. The DIXON method segments the tissue only into soft tissue and adipose tissue. Bone is classified as soft tissue, which underestimates attenuation. In UTE MRAC, the bone is segmented and assigned a corresponding attenuation coefficient. Due to slightly different MR properties of gypsum compared to bone, the gypsum might not have been completely recognized as bone (cf. Fig. 2). For this reason, segmentation leads to an underestimation of the bone component, which in turn leads to an erroneous estimation of the attenuation.

Not all human structures and tissues can be imitated in a handmade phantom as presented here. This might lead to errors with atlas-based MRAC methods, such as PSEUDO-CT [16], because the registration of the MR images of the phantom to the stored templates does not work as intended due to the missing structures.

The effective $R^{\star }_{2}$ for cortical bone as obtained from the UTE data of patients is lower than expected for pure cortical bone [13]. This is probably due to partial volume effects which are exacerbated by the fact that the water concentration in bone is less than in the surrounding tissue. MR contrast agents might be used to increase $R^{\star }_{2}$ of plaster to match that of cortical bone. However, both materials, cortical bone and plaster, show the same effective $R^{\star }_{2}$ as seen by the UTE sequence. Hence, the UTE-based MRACs accept plaster as a substitute for bone.

Small air inclusions are visible in the gypsum which can lead to susceptibility artifacts in MRI. Therefore, care should be taken to keep air inclusions to a minimum when creating the bone imitation. This problem can also occur during processing of the agarose gel. The agarose gel hardens as it cools down and shrinks, causing cracks that trap air. These should be filled subsequently.

The use of iodine to adjust the attenuation of gypsum can cause mistakes in the bilinear scaling of AC in PET-CT because relative attenuation is higher at CT photon energies than at 511 keV of PET. However, as listed in Table 2, the attenuation coefficients obtained by PET-CT and the PET transmissions scan are very similar so that a major error caused by iodine can be ruled out. Furthermore, iodine would lead to an overcorrection of attenuation in PET-CT which is not observed in this study (cf. Fig. 3).

Concerning the substitute for adipose tissue, more work is certainly needed to adjust the PET and MR properties to match those of the genuine tissue: Firstly, the short $T^{\star }_{2}$ of silicone needs to be addressed. As this short $T^{\star }_{2}$ is a result of the polymeric structure of hardened silicone, a more liquid substance is required which imitates the lipid inside the adipocytes of adipose tissue. Lipid-filled compartments (e.g., macroscopic compartments in the phantom or microscopic spheres) might be possible candidates to create such a material. Secondly, the attenuation coefficient of one-component silicone is remarkably higher in PET-CT compared to that obtained in the PET transmission scan (cf. Table 2). Also, the attenuation coefficient is higher than that of water for PET-CT. However, the mass density is lower for silicone (samples of cured silicone swim in water). One reason for this deviation might be errors in the bilinear scaling used for AC in PET-CT caused by the different atomic composition of the material (e.g., silicon). An additional task of the silicone is to keep the phantom sealed and in shape (Fig. 1). This has to be considered when replacing the silicone with another material. The need for a better replacement of adipose tissue will become more important for realistic abdominal phantoms where the impact of attenuation due to fat on the PET images is higher.

In order to minimize the errors of atlas-based methods, such as PSEUDO-CT, more attention should be paid to imitate the anatomical proportions. For example, the skin structures and the proportions of adipose tissue in particular are not modeled in sufficient detail by the phantom presented. Moreover, tissue-air interfaces in the nasal cavities, which are challenging for UTE-based MRAC methods, could be modeled by including air-filled balloons (analogous to the trachea and esophagus as described above) in order to assess the performance of these methods. The flask placed in the phantom allows only for a simple homogeneous activity distribution. In order to simulate more complicated physiological activity distributions, the structure of the brain could be imitated in more detail.

3D-printing techniques could be an alternative when creating the bone-imitating parts of the phantom, either by creating the bone imitations directly or by creating impression molds. For long-term stability, chemical agents could be added to keep the phantom sterile.

As mentioned above, the aim of this work was to test the hypothesis that building a suitable PET-MRI phantom is possible, i.e., whether it is generally possible to build such a phantom before going into detail about more sophisticated substances or detailed anatomical structures. To pass this test, a phantom should provide satisfactorily correct global quantification results for a variety of MRAC methods. This would allow using it for other purposes such as investigating the efficiency of MR-based motion correction of PET data with minimum interference from MRAC issues. Hence, it was not the primary intention of this study to rate the performance of different MRAC methods at this stage. Rather, the various MRAC methods were used to test whether the phantom is suitable for different MRAC approaches. The comparison of activity concentration (brain phantom vs. sphere) was used as a measure for this suitability. To pass the test, the error in quantification should be on the same order of magnitude as that of PET-CT or other devices (blood sampling, dose calibrator). If, for example, the comparison would yield deviations far above 10% for one or more of the MRAC methods, this would indicate that a completely different approach is needed for PET-MRI phantom. As this was not the case, the phantom can serve as a prototype for further developments.

## Conclusions

The created phantom consists of three types of tissue substitutes: water-saturated gypsum (bone), one-component silicone (adipose tissue), and agarose gel (brain mass). The phantom adequately represents the human head in terms of its attenuation properties and MR characteristics. The comparison of the determined radioactivity concentration in the centrally placed flask with that of a spherical phantom to test several MRACs of the phantom showed relatively little error in quantification, comparable to that of PET-CT. This indicates that it is possible to construct a brain PET-MRI phantom that leads to MR-based attenuation-corrected images with reasonable accuracy.

## Data Availability

The datasets generated during and/or analyzed during the current study are available from the corresponding author on reasonable request.
